# A Double Lesion of the Brain—Cerebral Cyst—Cerebellar Tumor1Read at the meeting of the Neurological Section of the American Medical Association, Milwaukee, June, 6, 7 and 8, 1893.

**Published:** 1894-04

**Authors:** Edward H. Angell

**Affiliations:** 295 Alexander Street; Rochester, N. Y.


### A DOUBLE LESION OF THE BRAIN—CEREBRAL CYST—CEREBELLAR TUMOR.[1]

By EDWARD B. ANGELL, M. D., Rochester, N. Y.

Some months ago, a case of obscure brain trouble came under my care, the symptomatology of which readily enough determined a diagnosis of cerebellar tumor. Had this been the only morbid condition, the case would hardly have been of more than clinical value. But an earlier lesion, resulting in the destruction of certain important convolutions of the cerebrum, has some scientific bearing upon cerebral pathology, and the functional correlation between the two hemispheres. For this reason I desire to place the case upon record before this association.

Mary O., aged 32 years, single, a shoemaker by occupation, of good social status, family history negative; the personal history unimportant

---

1. Read at the meeting of the Neurological Section of the American Medical Association, Milwaukee, June, 6, 7 and 8, 1893.

with one exception. At the age of five years, during convalescence from a severe attack of scarlatina, sudden right hemiplegia developed, due doubtless to a thrombosis of a branch of the left mid-cerebral artery. It was attended with loss of speech, but without loss of consciousness. Of this early palsy the details will be given later.

Aside from defective development of the right arm and hand, it left little trace upon the constitution. She enjoyed good health till two and one-half years before her death, when the symptoms, due to the cerebellar tumor, gradually developed. Briefly noted in the order of their appearance they were, physical and mental languor, slight vertigo and later uncontrollable vomiting, unstable equilibrium, occasional occipital headache, some parasthesia in the right arm and leg, deafness of the right ear, later becoming complete. The cerebellar ataxia gradually increased, the tendency to fall being toward the right side and backward. About a year before death vision in the right eye failed and ultimately there was almost obliteration of special sensation. The intellect, however, was very little impaired, and that only on account of the pressure exerted by the encroaching tumor. There was no involvement of the third, fourth or sixth pair of nerves. Right optic neuritis was pronounced. The knee jerk was equally exaggerated on either side and increased by reinforcement. Occasionally the urine showed traces of sugar, but no albumin. Sensory response to pain, touch and temperature was well preserved to the end. Operative measures were not advised on account of the probably inaccessible location of the growth.

The autopsy revealed a nodular, encapsulated tumor—a gliosarcoma. It was the size of a small egg, spherical in shape, and weighed two ounces. It lay between the right cerebellar hemisphere and the inferior margin of the right temporo-sphenoidal lobe, with the occipital lobe above, and pressed directly upon the corpora quadrigemina. The growth was bounded on its inner side by the right crus cerebri, the pons and upper portion of the medulla. These last two structures had been crowded inward by the tumor, and thus several of the cranial nerves springing from them had been subjected to pressure. The growth was almost entirely free from the surrounding organs, although it involved some of the cerebellar tissue. It sprung from the middle peduncle and was partly covered by the cerebellar convolutions. It was detached enough for ready removal, but its position anterior to the cerebellum and adjacent to the most vital structures of the brain rendered it inaccessible. Of the cranial nerves only the auditory and glosso-pharyngeal were subject to much pressure, although the trigeminal was somewhat involved.

Upon removing the calvarium, the membranes were found unaltered and the blood-vessels uncongested. The cerebral convolutions, though flattened, seemed normal, and there was no superabundance of serous fluid. But just above the junction of the fissures of Sylvius and Rolando was a cyst, grayish in color and filled with serous fluid. This cavity, the remnant of the destructive thrombosis of early childhood, was limited below by the ascending and horizontal limbs of the Sylvian fissure, and reached down to the sheath of the left ventricle—the external capsule and claustrum. It replaced the lower precentral convolution, the upper part of the operculum, a narrow strip of the ascending parietal along the Rolandic fissure and nearly the posterior half of the island of Reil, practically almost the whole of the speech center. Not only had specific convolutions been destroyed, but the whole hemisphere suffered arrest of development. The left cerebrum weighed sixteen ounces, four ounces less than the right, and its convolutions were less prominent, its sulci shallower and the cortical gray matter thinner.

Section of the brain showed a similar atrophy of the basal neuclei. The left striate body was only three-fourths the size of the right, while the left optic thalamus was a third smaller than its fellow. The atrophy of the left crus cerebri was marked and extended into the pyramidal tract.

The right cerebellar hemisphere was a fifth smaller than the left, weighing two ounces, while the left weighed two and one-half ounces. The variation in size unquestionably was not due to the cerebellar tumor, as might be supposed, but rather to the earlier defect; since in atrophy of one cerebral hemisphere, the alternate cerebellar hemisphere, especially its middle lobe, is retarded in development. This proportional loss in the right cerebellar hemisphere was exactly identical with that of the left cerebral—in either case the weight being twenty per cent. less than that of its fellow.

Curiously the cerebral convolutions of the right side, corresponding to the ones destroyed in the left, were strikingly prominent in comparison with the other convolutions of the same side, or with similar convolutions in other brains. The Rolandic convolutions were in reality hypertrophied, and anatomically suggested unusual functional activity.

Outside the brain, as was to be expected, there was more or less unilateral atrophy of the tissues. The lesion had nearly destroyed the right arm area, as well as those of the face and tongue. The leg area had not been invaded, consequently its growth was but little retarded. But the right arm and hand were very small and almost useless, while the right side of the face was more *petite* than the left, and that half of the tongue materially thinner and less prominent.

Careful questioning established these facts. Before the paralysis she was right-handed; after recovery she slowly became left-handed, learning to write with that hand, though poorly and always from right to left. The paralysis lasted about three months. There was complete loss of speech for four or five weeks; then it was gradually restored, though two months more were required before she could talk well. Her mother volunteered the remark that "she seemed to learn to talk over again." There was no noticeable mental impairment subsequently, though she was not an apt scholar and cared little for study. Memory was always good, her temperament cheerful and contented. She possessed a strong will power.

In her movements she was rather awkward; could not learn to dance, found it difficult to run, and in other ways showed evidence of defective muscular action.

This case of acquired porencephaly possesses two features of some importance. We know that a lesion of the left third frontal convolution with consequent loss of speech, is soon compensated in early life by the development of a speech function which apparently lies dormant in the corresponding convolutions of the opposite hemisphere. But in the present instance, in addition to the clinical facts, is the anatomical one of undue development of the right motor convolutions corresponding to the ones destroyed on the left side. Again, this decided hypertrophy of certain cerebral convolutions, due to increased functional need, exemplifies the effect muscular activity exercises upon the growth of the controlling nervous centers. Is it not fair to assume that we may thus explain, in part, the value of passive muscular exercise, whether by electricity, massage or other means, in the treatment of paralysis? Mechanical stimulation of the affected muscles not alone benefits by improving local nutrition. It has central influence as well. I believe that persistent exercise of any muscle distinctly influences the growth of its cortical center. It would be a matter of great value to determine whether physical training, influencing as it must the motor area of the brain, stimulates in any way intellectual development.

295 Alexander Street.

